# The Topography of Titanium in Dental Implants: Key to Osseointegration and Bactericidal Capacity

**DOI:** 10.3390/ma18143368

**Published:** 2025-07-17

**Authors:** Marta Romero, Manuel María Romero-Ruiz, José Vicente Rios-Santos, Blanca Rios-Carrasco, Mariano Herrero-Climent, Aritza Brizuela-Velasco, Jordi Martínez-Lopez, Javier Gil

**Affiliations:** 1 Departamento de Periodoncia, Facultad de Odontología, Universidad de Sevilla, c/Avicena s/n, 41009 Sevilla, Spain; martars.96@gmail.com (M.R.); mmromero@infomed.es (M.M.R.-R.); jvrios@us.es (J.V.R.-S.); brios@us.es (B.R.-C.); 2 Porto Dental Institute, Av. de Montevideo 810, 4150-518 Porto, Portugal; dr.herrero@herrerocliment.com; 3 DENS-ia Research Group, Faculty of Health Sciences, Miguel de Cervantes European University, 47012 Valladolid, Spain; aritzabrizuela@hotmail.com; 4 Soadco Research and Development Department, AD700 Escaldes, Andorra; j.martinez@soadco.com; 5 Bioinspired Oral Biomaterials and Interfaces, Departamento de Ciencia e Ingeniería de Materiales, Escola d’Enginyeria Barcelona Est, Universitat Politécnica de Catalunya, Av. Eduard Maristany 16, 08019 Barcelona, Spain

The following five factors established by Albrektsson for osseointegration of dental implants are well known: dental implant material, design, surface, surgical technique, bone quality, and mechanical conditions [1,2]. All these factors have been studied in depth to obtain dental implants with high osseointegration capacity even in adverse situations, such as in patients with poor bone quality or severe mechanical loads [3,4].

Dental implants are currently made of commercially pure titanium and titanium alloys such as Ti6Al4V or Ti13Zr. Zirconia ceramic dental implants are also used because, although they are more brittle than metal implants, they have better esthetics, and there are no problems of corrosion or allergies, although there are very few references due to these problems [5,6]. One of the current lines of research is aimed at achieving a dental implant with a modulus of elasticity more similar to human bone (10–20 GPa), since titanium and its alloys have higher values of around 110 GPa [7]. This difference causes the transfer of mechanical load to the bone to not be the most adequate, causing bone resorption. In order to reduce these differences in the elastic modulus, low modulus or beta-titanium alloys are being investigated, incorporating elements such as zirconium, hafnium, and niobium, which make it possible to reduce the values to 40 GPa. It has been shown that the closer the modulus of elasticity of the metal is to the bone, the greater the osseointegration, and the bone resorption is reduced by about 70% [8].

A very interesting study was the implantation in minipigs of dental implants with the same design, one of them made of zirconia, another of commercially pure titanium with a smooth surface, and the third group of commercially pure titanium with an Sa-roughness of 1.3 μm. They were implanted for 4 and 12 weeks. The histologies showed that the smooth titanium and zirconia dental implants had very similar bone implant contact (BIC) values of 24 and 32% in four weeks, respectively, and the rough one reached a value of more than 45%. At 12 weeks after implantation, the BIC values increased to 41 and 40% for zirconia and smooth titanium, respectively, and rough titanium reached values of 74%. This fact highlights the importance of roughness in osseointegration and confirms the limitation of ceramic implants since, due to their fragility, they cannot offer roughness obtained by projection of abrasive particles. The impacts of the abrasive particles on ceramic could generate cracks on the surface, which, with the cyclic chewing load, would propagate until catastrophic rupture. Acid attacks are very ineffective since the ceramic is very inert and the roughness obtained is very small [9,10].

It has been shown that osteoblast adhesion is very sensitive to roughness, and it can be affirmed that topographies with an Sa of 0.9 to 1.4 μm are optimal values for osteoblastic activity. Different ways of obtaining this roughness have been studied, such as acid etching, titanium plasma spraying, or grit-blasting, among the most important [11,12]. It has been confirmed that acid etching offers too small a roughness because of the influence of the titanium grain size, and the plasma spray technique does not allow for optimizing the roughness, and its economic cost is very high. However, grit-blasting makes it possible to obtain the desired roughness since we can vary the nature of the abrasive particle that will impact the titanium, its size, the projection pressure, the distance from the surface to the gun, and its diameter. The optimization of these parameters allows us to optimize the surface roughness [13].

In most dental implants, alumina (Al_2_O_3_) is used as an abrasive agent. It is not suitable to use titanium oxide (TiO_2_) because it does not have the abrasive capacity since this oxide is of the same nature as the substrate and produces very little roughness [14]. However, alumina causes a plastic deformation in titanium and its alloys that allows having the appropriate topography. Several authors were concerned that aluminum oxide particles could anchor on the implant surface, acting as inhibitors of bone mineralization [15]. This claim has been proved to be false. Alumina grit-blasting tests were carried out on dental implants; some were left with residual alumina, which, in general, is 8% alumina on the surface, and others with the same roughness were washed with high-power ultrasound to remove any residual alumina. It was found that dental implants with alumina residues had higher osteoblastic activity. Also, the results of in vivo tests showed, for the same implantation time in minipigs, a higher BIC value [16,17]. The authors explain this behavior with physicochemical properties since the alumina residues make the surface more hydrophilic and with higher surface energy, especially in its polar component, which increases the adsorption of osteoblast adhesion precursor proteins. Dental implants with aluminum oxide have a higher negative charge on the surface due to the three oxygens that form alumina and therefore favor the adsorption of fibronectin [18]. The results also show that the traces of alumina have a slight bactericidal character due to the oxidizing capacity of this oxide on the surface [16].

Not only does grit-blasting treatment provide the optimum roughness for osteoblastic activity, but it also improves the fatigue mechanical properties. When the treatment of projection of abrasive particles on the titanium surface causes plastic deformation, producing roughness but also creating a layer of internal tension on the surface of compressive character. This stress inhibits the formation of surface cracks and delays the appearance of the crack due to the chewing cycles. The increase in fatigue limit between samples without grit-blasting and with the treatment is more than 25%. At normal masticatory loads, the number of fatigue cycles exceeds 20 million cycles and can be considered as implants with infinite life when treated with grit-blasting [19,20].

On the other hand, recent studies have shown that compressive residual stress on the titanium surface reduces the contact angle by 20 to 30° compared to implants without compressive residual stress [21]. This increase in hydrophilicity leads to an increase in osteoblastic activity. This fact is corroborated by in vivo tests, where implants of equal roughness and design with and without compressive residual stress were inserted in rabbit tibiae, showing that the tensioned implants presented a BIC of more than 15%, with respect to the implants without tension. Therefore, it could be considered that the tensioned state of the dental implant is a factor that facilitates osseointegration and could be added to Albrektsson’s list. In any case, stressed implants show an increase in bacterial proliferation, with respect to unstressed implants [21].

Bacteria that act in the mouth can form biofilms and can generate peri-implantitis. This disease causes 24% of dental implants to fail within 10 years of placement. These figures are very alarming, and it is for this reason that microbiological research has increased in an attempt to reduce these failure rates [22]. Studies have been carried out on the influence of roughness on bacterial colonization, and it has been determined that the greater the roughness, the greater the number of colonies. Romero et al. have used titanium discs with 16 different roughnesses from 0.05 to 5 μm of Sa and have been able to determine the increases for 2 common bacterial strains in the formation of biofilms in the oral cavity [23]. It has been possible to verify how there is a linear growth from 0.1 to 2 μm and there is a sharp increase in the slope from 2 to 5 μm of Sa. This fact makes it necessary for dental implant manufacturers to regulate the roughness in a way that is optimal for osteoblastic activity and the worst possible for bacterial colonization. A remarkable aspect of some research is that the polished surface is not the one that offers minimum values of microbiological activity, but there is a very fine roughness of 0.05 to 0.07 μm that generates peaks on the surface that are able to penetrate the membranes of bacteria and cause their death, inhibiting or at least hindering the formation of biofilms [24].

Recently, hybrid dental implants have appeared in which the coronal part has a polished surface to hinder bacterial colonization, and the apical part has rough titanium to improve osseointegration. These implants present an important controversy since they sacrifice osseointegration to make biofilm formation more difficult. Moreover, in the rough-polished border areas, they may be more susceptible to the onset of fatigue cracks or cause a decrease in corrosion resistance [25].

It has been proven that antibiotic treatment is not the most appropriate to prevent long-term infection, since they have a temporary effect, and in many cases their action does not exceed two weeks, and there is a possibility of resistance, among the most important limitations. It is for this reason that it is necessary for the conventional titanium oxide passivation layer to be converted into a bactericidal or at least bacteriostatic layer. In this sense, there are different lines of action, such as the formation of biofunctionalized titanium surfaces [26].

Biofunctionalization consists of reacting the titanium surface by means of Piranha reagents and silanization processes that allow organic molecules, amino acids, peptides, or proteins with bactericidal capacity to be attached to the titanium. Biofunctionalization with lactoferrin is an example of a highly effective bactericidal surface [27].

It is also known that the bacteriostatic effect of polyethylene glycol can rebound titanium and can even serve as a platform to biofunctionalize titanium with bactericidal agents. There are also authors who have demonstrated the ability to bind organic chains that help cellular activity, such as RGD and bactericidal chains, producing three-in-one structures on surfaces: bacteriostatic (PEG), cellular activity (RGD), and bactericidal (lactoferrin) [28]. This is undoubtedly a very promising way to achieve surfaces with different purposes.

Likewise, passivation agents such as citric acid and EDTA, among the most important ones, have been studied, which generate an oxidizing character on the titanium surface that causes the death of numerous types of bacterial strains, both Gram + and Gram − [29,30,31,32]. The latest studies based on passivation with sulfuric acid and hydrogen peroxide solutions create on the titanium surface a titanium dioxide structure with nanopillar morphology that has a great bactericidal capacity due to the penetration of the nanospikes through the membranes of the different bacteria. These results are very promising, and studies have been carried out on different biofilms commonly found in the oral cavity, showing a significant reduction in bacterial activity of more than 70% [33]. It was found that this nanotopography does not affect cell viability since the size of the cells is much larger than the bacteria and the nanospikes go unnoticed by them. This surface does not affect the mechanical properties and makes the surface more hydrophilic, which also leads to an improvement in the biological activity of the titanium [34,35,36,37]. In vivo studies of this surface on rabbit tibiae gave results of 7 to 10% higher BIC for nanospikes than for conventional passivation-coated implants [33].

As we have seen in this Special Issue, the surface of titanium, whether used as a dental implant or as a prosthesis, can be treated to achieve properties that favor the good behavior of the implant system. There are many strategies to be addressed in the future in order to achieve long-term reliability of dental implants and prostheses to fulfill the mission of tooth replacement with adequate functionality and esthetics.
